# A Priori Data-Driven Multi-Clustered Reservoir Generation Algorithm for Echo State Network

**DOI:** 10.1371/journal.pone.0120750

**Published:** 2015-04-13

**Authors:** Xiumin Li, Ling Zhong, Fangzheng Xue, Anguo Zhang

**Affiliations:** 1 Key Laboratory of Dependable Service Computing in Cyber Physical Society of Ministry of Education, Chongqing University, Chongqing 400044, China; 2 College of Automation, Chongqing University, Chongqing 400044, China; Tianjin University, CHINA

## Abstract

Echo state networks (ESNs) with multi-clustered reservoir topology perform better in reservoir computing and robustness than those with random reservoir topology. However, these ESNs have a complex reservoir topology, which leads to difficulties in reservoir generation. This study focuses on the reservoir generation problem when ESN is used in environments with sufficient priori data available. Accordingly, a priori data-driven multi-cluster reservoir generation algorithm is proposed. The priori data in the proposed algorithm are used to evaluate reservoirs by calculating the precision and standard deviation of ESNs. The reservoirs are produced using the clustering method; only the reservoir with a better evaluation performance takes the place of a previous one. The final reservoir is obtained when its evaluation score reaches the preset requirement. The prediction experiment results obtained using the Mackey-Glass chaotic time series show that the proposed reservoir generation algorithm provides ESNs with extra prediction precision and increases the structure complexity of the network. Further experiments also reveal the appropriate values of the number of clusters and time window size to obtain optimal performance. The information entropy of the reservoir reaches the maximum when ESN gains the greatest precision.

## Introduction

Echo state networks (ESNs), proposed by H. Jaeger in 2004 [1], have attracted a great deal of attention because of its high accuracy, fast learning speed, and global convergence [2]. ESNs have been widely applied to practical applications, such as time series prediction [3, 4], classification [5, 9], and anomaly detection [6]. These superiorities are attributed to a large and fixed dynamical system called dynamical reservoir (DR). Adaptation is treated as a simple linear regression problem because of the sufficient richness of DR. Calculation is also greatly simplified.

The reservoir, being a key part of ESN, is always a focus of research. Various network topologies of DR have been investigated [7–9] because of the performance of ESN being mainly determined by the DR structures [10]. The construction of DR with complex structure has been widely investigated based on the complex network theory [11–15]. Among these structures, the multi-cluster structure has attracted significant attention because of its rich dynamics and bionic characteristics, such as small-world reservoir [16], critical reservoir [17], and modular reservoir [7]. However, the corresponding ESNs are affected by complicated structure factors although networks with this structure have a powerful calculating ability. These factors include cluster number and size and connection form of the intra- and inter-cluster [11]. The performance of an inappropriate multi-cluster structure is worse compared with that of conventional ESN. Researchers have started to study the establishment of an ideal multi-cluster reservoir to solve the preceding problem (i.e., generating problem of multi-cluster reservoir). Two methods are currently employed to construct the multi-cluster reservoir. The first method is a simple spatial growth algorithm [18], where the probability for edge formation depends on the spatial distance between nodes. However, the model parameters could not control the cluster size; even multiple clusters are not guaranteed [17]. The second algorithm is a cortex-like network generation method based on the spatial distance between nodes and the associated time window [19], which overcomes the preceding deficiencies. However, the existence of randomness in this generating algorithm leads to a very limited precision improvement and a largely fluctuating performance [11].

Finding a universal solution for reservoir generating problems is difficult because the network cannot be evaluated before application. However, sufficient priori data exist in some application areas (e.g., International Road Traffic and Accident Database [20], Electricity Data Browser [21], and Global Financial Development Database [22]). These data can be used to design an analog application in advance to evaluate and construct a multi-cluster reservoir. Furthermore, a novel iterative generating algorithm for a multi-cluster reservoir can be designed to guarantee the final reservoir performance to some extent. These priori data are performance-oriented with regard to reservoir construction. This generation algorithm is called a priori data-driven generation algorithm. The reservoir is established by repeated evaluations of the priori data. The corresponding ESN should exhibit an excellent performance because these data are the recognized authority in this application field.

This study proposes a novel ESN based on the priori data-driven multi-cluster reservoir generation algorithm (DDMCESN). A benchmark prediction task (i.e., Mackey-Glass chaotic time series (MGS) prediction) is employed as the network performance-testing platform without loss of generality. The comparison experiment of the network performances based on this platform confirms the advantages of the DDMCESN over the ordinary multi-cluster ESN (MCESN) and the traditional random ESN. The results prove the effectiveness of the proposed network generation algorithm. The effects of some structure parameters on the network performance are also discussed.

Section 2 briefly presents the ESN principle and describes the DDMCESN. Section 3 examines the prediction of the Mackey-Glass time series to analyze the characters and performances of the ESN with a modified cluster structure. Section 4 presents the discussion and the conclusion.

## Reservoir Construction of DDMCESN

### Network Structure of ESN

**Fig 1:**
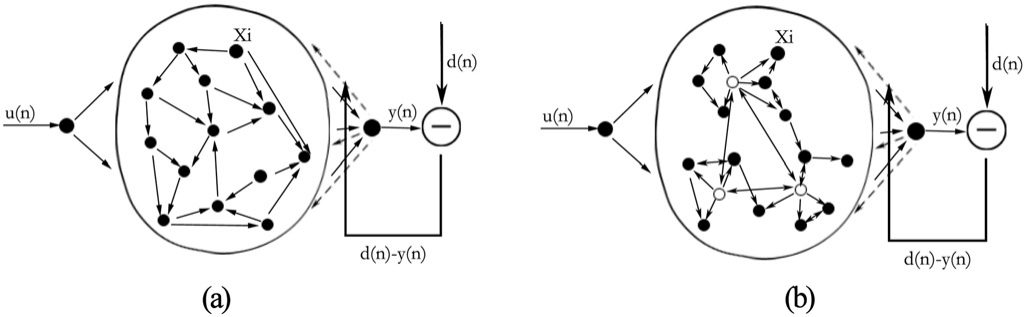
System architecture: (a) scheme diagrams of the conventional random ESN and (b) DDMCESN models.

The architecture of ESN comprises an input layer, dynamical reservoir, and readout neuron. The conventional ESN has a randomly connected reservoir with large-scale neurons Fig 1(a). The DDMCESN has a reservoir with a multi-cluster topology unlike the conventional ESN Fig 1(b). The ESNs are assumed to have K input neurons, N reservoir neurons, and L readout neurons. The connection weights from the reservoir neurons to the readout neurons are given in a **N∗K** matrix **W**_*in*_. The reservoir connection weights are collected in a **N∗N** weight matrix **W**. The connection weights from the reservoir neurons to the readout neurons are given in a **L∗(K+N+L)** matrix **W**_*out*_. Furthermore, the connection weights projected back from the readout neurons to the reservoir neurons are given in a **N∗L** matrix **W**_*fb*_. The reservoir is updated according to the following equations given a training sequence of **y**_*d*_(*n*), *n* = 1, 2…*T*

$$\begin{array}{c} x\left(n+1\right)=f(W_{in}u\left(n\right)+Wx\left(n\right)+W_{fb}y_{d}\left(n\right))\cdot \end{array}$$ (1)

$$\begin{array}{c} y\left(n+1\right)=f_{out}\left(W_{out}\left(u(n+1),x(n+1),y(n)\right)\right), \end{array}$$ (2)

where **x**(*n*) represents the state variables in the reservoir; **u**(*n*) and **y**(*n*) are the input and the output of ESNs, respectively; **f** and **f**_*out*_ are the hyperbolic tangent function applied componentwise.

Weight matrices **W**_*in*_ and **W**_*fb*_ are drawn from a uniform distribution over [−1, 1]. **W**_*out*_ is the only one trained by the ESN training algorithm. Furthermore, the weights are arbitrary values usually initialized by 0. **W**_*out*_ is computed via pseudo-inverse as follows:

$$\begin{array}{c} P=E[x(n)],T=E[d(n)], \end{array}$$ (3)

$$\begin{array}{c} W_{out}={\mathrm{TP}}^{+}, \end{array}$$ (4)

where **x**(*n*) represents the internal states of the reservoir at time *n*, and **d**(*n*) is the desired output signal at time *n*.

### The priori data-driven strategy for multi-cluster construction

A priori data-driven multi-cluster reservoir generation algorithm is proposed to establish a suitable multi-cluster reservoir. The algorithm strategy comprises three steps: generation of an initial multi-cluster reservoir, evaluation and update of reservoirs, and termination conditions of the reservoir update. These three procedures are discussed as follows:

1) Generation: A multi-cluster reservoir is generated according to Kaiser’s clustering algorithm [19]. Two growth mechanisms, namely, spatial distance between nodes and associated time windows, manage the generating process. A connection between neurons **U** and **V** made on time step *t* is established with probability as follows:

$$\begin{array}{c} P=P_{dist}\left(d\left(u,v\right)\right)*P_{time}^{\mu_{u}}\left(t\right)*P_{time}^{\mu_{v}}\left(t\right), \end{array}$$ (5)

where **P**_*dist*_(*d*(*u*, *v*))) = *βe* ^−*αd*(*u*, *v*)^ is the distance-dependent probability; *d*(*u*, *v*) is the Euclidean distance between neurons **U** and **V**; and *α* and *β* are the scaling coefficients shaping the connection probability (i.e., *α* = 6 and *β* = 6). The time window-dependent probability is computed as follows:

$$\begin{array}{c} P_{time}^{\mu_{i}}\left(t\right)=P\left(t;i,\varphi \left(\mu_{i},\omega \right)\right)=\frac{1}{16}{\left(t^{2\lambda }{\left(t^{\lambda }-1\right)}^{2}\right)}^{1/\varphi (\mu_{i},\omega )}\cdot \end{array}$$ (6)

where *t* = *j*/*N*, *j* = 1, 2, …*N*; *μ*_*i*_ = *i*/*n*+1, *i* = 1, 2, …*n*, where *n* is the number of pioneer nodes; *ω* is the time window integral value; *λ* = −(log(2)/log(*μ*_*i*_)); and *φ*(*μ*_*i*_, *ω*) is a numerically determined scaling factor used to compute the desired *ω* value.

2) Evaluation and update: Further adjustments are made to the structure based on the preliminarily obtained multi-cluster reservoir. These adjustments are necessary to improve the performance. The priori data during the reservoir update process used to be performance-oriented. Moreover, only the current reservoir with a better evaluation performance takes the place of the former. The prediction precision with a normalized root mean square error (NRMSE) and its standard deviation *δ* used as performance evaluations is employed to improve the calculating ability of the ESN. The updated reservoir is reserved if it has a smaller NRMSE or *δ*. The reservoir should be skipped and recovered to the former, otherwise.

3) Termination. The final reservoir is decided when the NRMSE reaches the preset requirement. The terminal condition is not simply set as a certain NRMSE value because the value is difficult to determine and varies with tasks and parameter settings. The reservoir update of the DDMCESN in this paper is terminated when the sum of the latest ten NRMSEs is smaller than the value of three-tenths of the sum of the previous ten NRMSEs.

### Multi-cluster reservoir generation algorithm based on priori data-driven strategy

The reservoir processing of the DDMCESN is implemented by the following steps based on the priori data-driven strategy in Section 2.2:

Step 1: The reservoir is generated from a small number of pioneer neural units, *n*. A 1∗1 plane area is selected, in which the *n* pioneer nodes are uniformly arranged. These *n* pioneer nodes are bidirectionally all-to-all connected to each other. The coordinates of unit *i* are described in Eq (7) as follows:

$$\begin{array}{c} x_{i}=\frac{i}{n+1},y_{i}=1-\frac{i}{n+1} \end{array}$$ (7)

Step 2: The new node **U** is randomly placed. This node corresponds to the nearest pioneer node. This node is also connected to the existent nodes according to Eq (5). A new node, which fails to establish a connection, is given up. Step 2 is repeated until the number of existing nodes reaches **N**.

Step 3 Each node connects itself with the self-connecting probability **P**_*s*_ after the network is created.

Step 4: The nodes of the generated network are mapped to obtain a multi-cluster reservoir. Matrix **W** reflects the connection strength between nodes. The nodes belonging to the same clusters are distributed together to simplify the analysis. The form of reservoir matrix **W** is rewritten as follows:

$$\begin{array}{c} W=(\begin{array}{cc} W_{1} & L_{1}\\ \ldots & \ldots \\ L_{2} & W_{n} \end{array}) \end{array}$$ (8)

where **W**_*n*_ is the connection matrix of the *n*th cluster; and *L*_1_ and *L*_2_ are the connecting matrices among different clusters. **W** theoretically presents the intensive diagonal because the connections among different clusters are relatively sparse.

Step 5: A stack with a length of 20 (**S**[20]) is defined, and a maximum iteration is determined using **Q**/10, where **Q** is the number of non-zero values in **W**.

Step 6: Accordingly, ten connections are randomly selected among the reservoir neurons. These connections are broken to product new internal weight matrix **W**. Subsequently, 10 repeated trials using the priori data are conducted to calculate the average NRMSE and *δ*. The current average NRMSE is then placed at the top of stack (**S**(1)) with the previous values moved backward.

Step 7: The updated **W** is compared with the previous one. Internal weight matrix **W** is preserved if its average NRMSE or *δ* is smaller than that of the previous one. The new **W** is undesirable, and the broken connections in Step 6 are recovered, otherwise.

Step 8: Whether current internal weight matrix **W** is ideal is determined. The reservoir network is generated and current **W** is maintained as the internal weight matrix of the DDMCESN if [*S*(1)+*S*(2)+…+*S*(10)]/3 < [*S*(11)+*S*(12)+…+*S*(20)]/10. Notably, the current prediction result cannot only be compared with the previous one because of the existing fluctuation. Step 7 is repeated until the terminal condition mentioned above is met or the iterations reach to the maximum.

## Experiment design and result analysis

This section examines the performance of the DDMCESN for a benchmark prediction task, that is, the 84-step prediction of the MGS. The effects of the cluster number and time window size on the performance of the DDMCESN are initially investigated. Comparison experiments are then designed to verify the effectiveness of the proposed algorithm. The performances are evaluated using prediction accuracy, information entropy, average shortest path, and clustering coefficient.

### Experiment design

The 84-step MGS prediction has become a benchmark prediction task for neural networks because of the richness of the MGS structure [23]. This study designs an 84-step MGS prediction experiment to analyze the network performances. The Mackey-Glass function is given in the following form:

$$\begin{array}{c} y\left[n+1\right]=y\left[n\right]+\delta (\frac{0.2y[n-\frac{\tau }{\delta }]}{1+y{\left[n-\frac{\tau }{\delta }\right]}^{10}}-0.1y\left[n\right]), \end{array}$$ (9)

**Fig 2:**
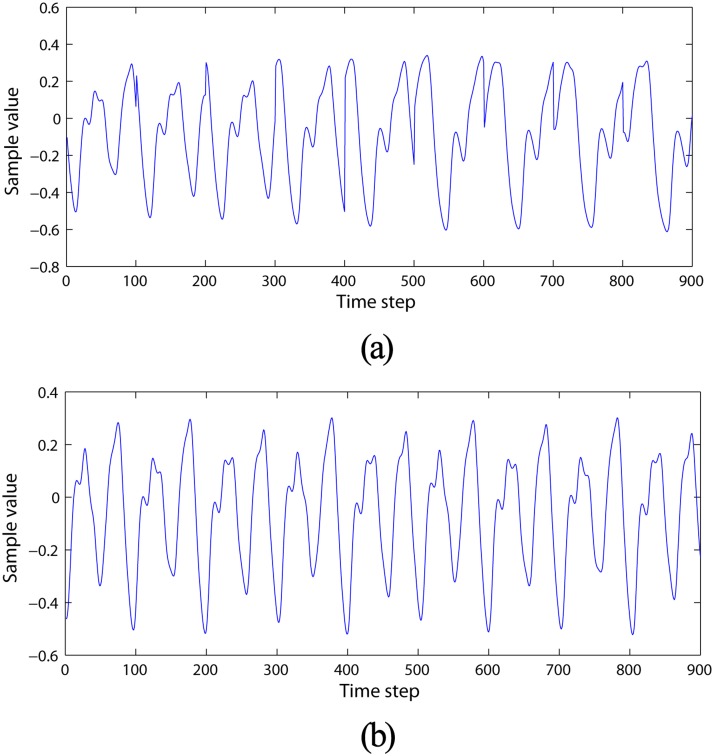
MGSs with 9000 sequences. (a) Prior data MGS for reservoir generation and (b) prediction target.

Where *δ* is the step size arising from the discretization of the original continuous-time Mackey-Glass equation. *τ* is a delay parameter influencing the chaotic degree of the MGS. A bias input of the same length is still created even though the MGS output does not depend on any input sequence. This bias input is simply used in the network state update procedure in Ref.[24]. The experiments comprise two stages, namely, reservoir establishment and MGS prediction by DDMCESNs. The MGS time series with chaotic dynamics is introduced as the priori data to guide the reservoir generation of the DDMCESN Fig 2(a). The system is chaotic when *τ* > 16.8, hence *τ* = [17, 18, 19, 20, 21, 22, 23, 24, 25] is chosen. The sample length of each case is 1000, and the total MGS length is up to 9000. The MGS with a 9000 length is created as the forecast target when the generated DDMCESN is applied to the MGS prediction task. A mildly chaotic behavior is produced when *τ* = 17 Fig 2(b). Accordingly, *δ* = 0.1 is always set with subsequent subsampling by 10. These MGSs are distributed in 4000 training and 5000 test sequences. The top 1000 samples are discarded to remove the initial transients.

The ESNs in the experiments have one input neuron with a fixed input *u* = 0.02 and a feedback (MGS) from the output sequence. The exact number of the reservoir neurons **N**, which are set to 400, is task-dependent. *ρ* stands for the spectral radius of the reservoirs. The *ρ* value should be smaller than 1 to ensure the echo state property (i.e., *ρ* = 0.8) [25]. Table 1 presents the other parameters.

**Table 1:** Parameter settings in the experiments.

**Parameter**	**values**
*α*	6
*β*	6
**P**_*s*_	0.5
**N**	400
*ρ*	0.8

Two test criteria are employed as the performance measurements in this simulation: prediction accuracy and its standard deviation. The prediction performance is measured using the normalized RMSE at the 84th time step(*NRMSE*_84_) and its standard deviation. The *NRMSE*_84_ is computed as follows:

$$\begin{array}{c} NRMSE_{84}=\sqrt{\frac{\sum_{i=1}^{l_{test}}{\left(y_{test}\left[i+84\right]-d_{test}\left[i+84\right]\right)}^{2}}{l_{test}\cdot \sigma^{2}}}, \end{array}$$ (10)

where *y*_*test*_[*n*] is the network output during the testing phase; *d*_*test*_[*n*] is the desired output during the testing phase; and *σ* ^2^ is the variance of the desired output. The standard deviation of *NRMSE*_84_ (*δ*) is defined as follows:

$$\begin{array}{c} \delta =\sqrt{\sum_{i=1}^{i=k}{\left(NRMSE_{84}\left(i\right)-NRMSE_{av}\right)}^{2}/k}, \end{array}$$ (11)

where $NRMSE_{av}=\sum_{i=1}^{i=k}(NRMSE_{84}(i))/k$, *k* denotes the number of independent trails.

**Fig 3:**
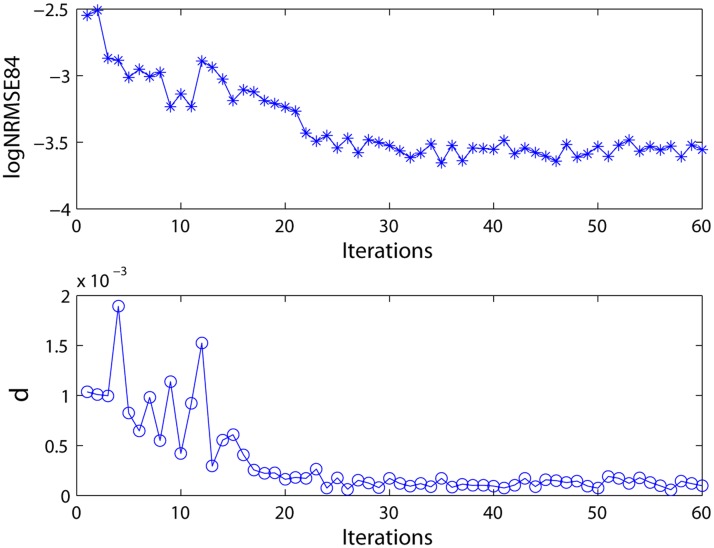
Prediction performance during the reservoir update. *NRMSE*_84_: normalized RMSE at the 84th time step. *δ*: standard deviation.

**Fig 4:**
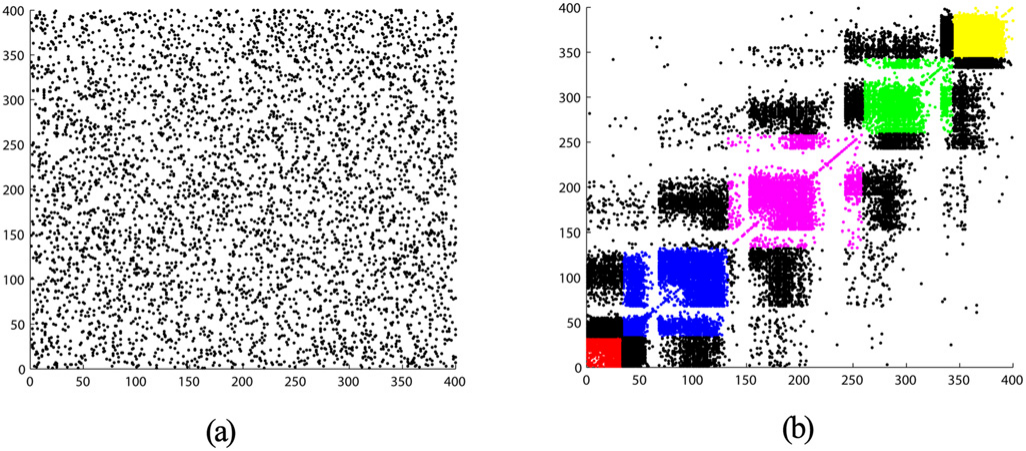
Structure matrix of the reservoir network for: (a) conventional ESN and (b) DDMCESN.

**Fig 5:**
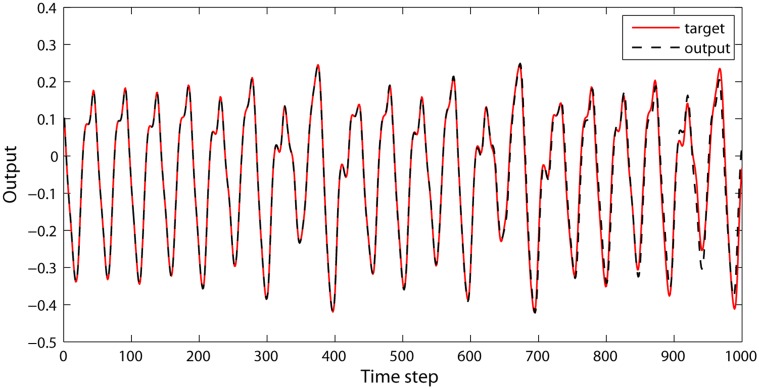
DDMCESN output in the test.

*NRMSE*_84_ and *δ* are calculated according to the priori data-driven multi-cluster reservoir generation algorithm described in Section 2.3 to guide the reservoir updating process and assess the current reservoir. The log_10_ *NRMSE*_84_ and *δ* values in this process are illustrated in Fig 3. The curves of both *NRMSE*_84_ and *δ* exhibit a decreased trend during the updating process (Fig 3), which indicates that the modeling capabilities of the reservoir tend to be enhanced. The connectivity structure of the DDMCESN reservoir is illustrated in Fig 4(b). The figure shows a scatter diagram of 400∗400 connection matrix **W** with *n* = 5 and *ω* = 0.3. The inter-cluster connections are represented by black spots, whereas the intra-cluster connections are described by colored dots. A clustering structure is obvious in Fig 4(b) unlike the random connectivity of the conventional ESN (Fig 4(a)). The intra-cluster connections are clearly more intensive than the inter-cluster connections. Moreover, the inter-cluster connections between the adjacent clusters are more concentrated than those between far clusters. A series of outputs is obtained using this reservoir matrix (Fig 5).

The number of clusters for the DDMCESN is controlled using the number of pioneer nodes *n*. The cluster size is varied by changing the width of the time window *ω*. The relationships between the prediction performance of the DDMCESN and the factors (*n* and *ω*) are investigated. The performances of the DDMCESNs compared with that of the MCESNs and the conventional ESNs are evaluated from three aspects. These aspects are the 1) prediction accuracy and its standard deviation; 2) information entropy of reservoir; and 3) average shortest path and clustering coefficient.

### Analysis of prediction accuracy

**Fig 6:**
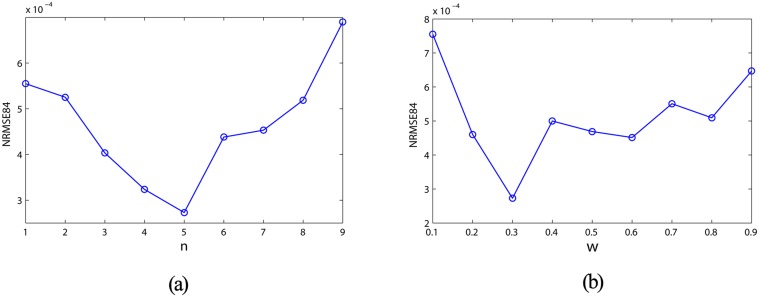
(a) Prediction accuracy of the DDMCESN influenced by the cluster size. (b) Prediction accuracy of the DDMCESN influenced by the time window size.

**Fig 7:**
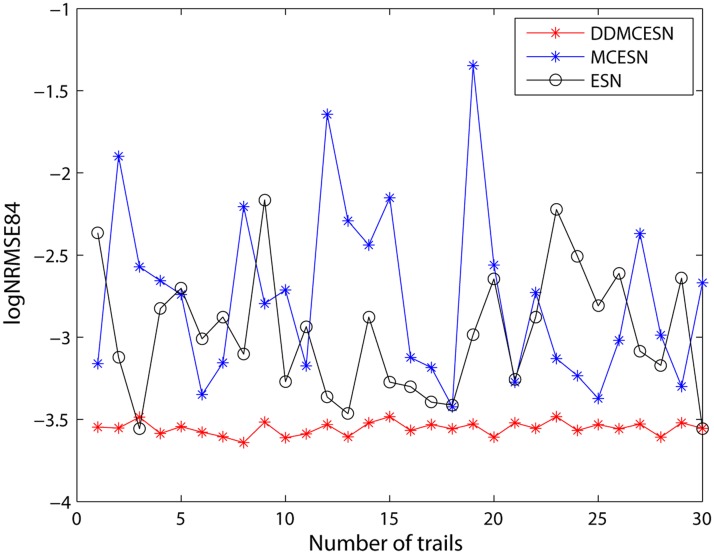
Comparison of the NRMSEs of ESN, MCESN, and DDMCESN with a cluster size of *n* = 5 and a time window size of *ω* = 0.3.

Prediction accuracy is an externally direct evaluation of the prediction problems, which reflects the calculating ability of the DDMCESN and affected by some parameters of the generating method. The relationship between the NRMSE and the clustering parameters (i.e., number of clusters and time window size) is first analyzed. Each case independently runs for 20 times. The *NRMSE*_84_ *s* reach the minimum values when the number of clusters is 5 and the time window size is 0.3 (Fig 6). A comparison of the prediction performances between the traditional ESNs, MCESNs, and DDMCESNs is performed in this case. As shown in Fig 7, MCESNs with multi-clustered reservoir show higher prediction accuracy than the conventional ESNs. Furthermore, due to the off-line refinement of the multi-clustered structure, DDMCESNs perform much better computing capabilities than the original MCESNs. The average values calculated through 30 independent and repeated trials are recorded in Table 2. The comparisons show that the DDMCESNs are significantly more excellent than the MCESNs and the conventional ESNs, indicating the effectiveness of proposed reservoir generation algorithm.

**Table 2:** Prediction performance of the three networks.

**Network**	***NRMSE*_84_**	***δ***
ESN	1.5∗10^−3^	0.0105
MCESN	9.7∗10^−4^	0.0017
DDMCESN	3.035∗10^−4^	0.00029

### Structure Complexity

Information entropy (H) is used to characterize the complexity of the synaptic connectivity distribution of the DDMCESN reservoir. Information theory states that entropy is a measurement of the uncertainty in a random variable [8, 26]. Entropy is defined as follows:

$$\begin{array}{c} H=-\sum_{i}P\left(x_{i}\right)\log(P\left(x_{i}\right), \end{array}$$ (12)

Where *P*(*x*_*i*_) is the probability that the average value of the synaptic strength *w*_*i*_ (normalized) lies within bin *k* (*k* ∼ (0, 1) with a step of 0.005).

**Fig 8:**
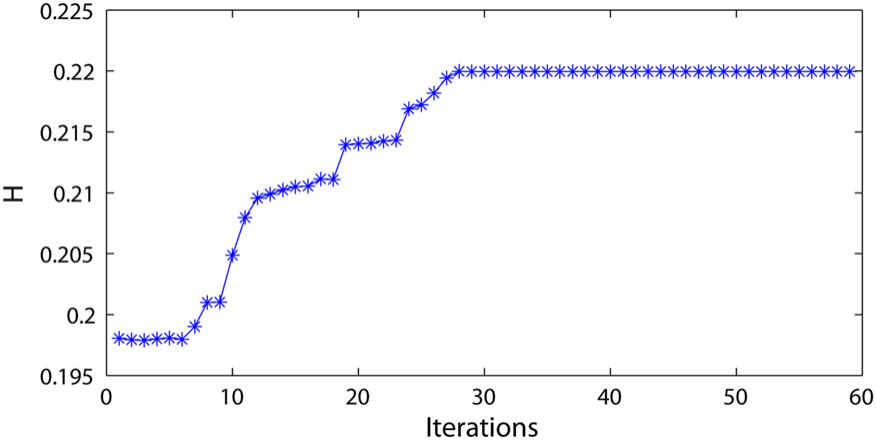
Entropy of the DDMCESN during the reservoir update.

**Fig 9:**
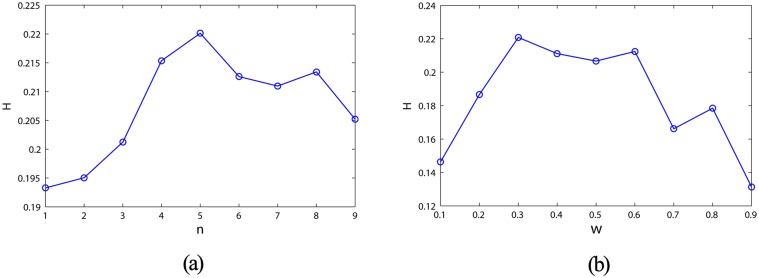
(a) Entropy of the DDMCESN reservoir influenced by the cluster size. (b) Entropy of the DDMCESN reservoir influenced by the time window size.

The information entropy of the network weight matrix during the reservoir update is recorded in Fig 8. The entropy gradually increases and ultimately remains unchanged when the weight matrix update becomes stable. The proposed generating method increases the structure complexity of the reservoir network. How the information entropy is influenced by the clustering parameters (i.e., *n* and *ω*) is illustrated in Fig 9. Accordingly, the entropy reaches the maximum in the case of *n* = 5 or *ω* = 0.3, where the DDMCESN has optimal accuracy (Fig 6). The results indicate that the highly complex structure of the reservoir network contributes to the computational capacity of the corresponding ESN network.

Table 3 presents the entropy of the three networks obtained by repeating the corresponding simulations for 30 times. The DDMCESN has a larger information entropy value than the others, which indicates that the reservoir network of the DDMCESN has the highest structure complexity. This complexity benefits from the computational performance of the reservoir computing.

**Table 3:** Entropy of ESN, MCESN, and DDMCESN.

**Network**	**DDMCESN**	**MCESN**	**ESN**
Entropy	0.220	0.213	0.179

### Average Shortest Path and Clustering Coefficient

The proposed DDMCESN networks with multi-cluster structure exhibit different topological properties from the conventional random network. We can investigate the inherent structure and dynamical characteristics of DDMCESNs reservoir in terms of the complex network theory. In particular, the average shortest path (ASP) [27] and the clustering coefficient (CC) [28] are introduced to characterize the small-world property of network with complex structure. ASP is the average number of links that have to be crossed to go from one node to another. ASP is calculated as follows:

$$\begin{array}{c} ASP=\frac{1}{N(N-1)}\sum_{i,j\neq i}d\left(i,j\right), \end{array}$$ (13)

where *d*(*i*, *j*) is the length of the shortest path between nodes *i* and *j*. The clustering coefficient (CC) of each node is the proportion of the direct links between the neighbors, quantifying the degree of its neighbors belonging to the same cluster [29].

$$\begin{array}{c} C_{i}=\frac{∥E(\Gamma_{i})∥}{\left(\begin{array}{c} k_{i}\\ 2 \end{array}\right)},CC=\frac{1}{N}\sum_{i}C_{i}, \end{array}$$ (14)

where |*E*(_*i*_)| is the number of edges in the *i* neighborhood, and $\left(\begin{array}{c} k_{i}\\ 2 \end{array}\right)$ is the number of possible edges of *i*.

**Fig 10:**
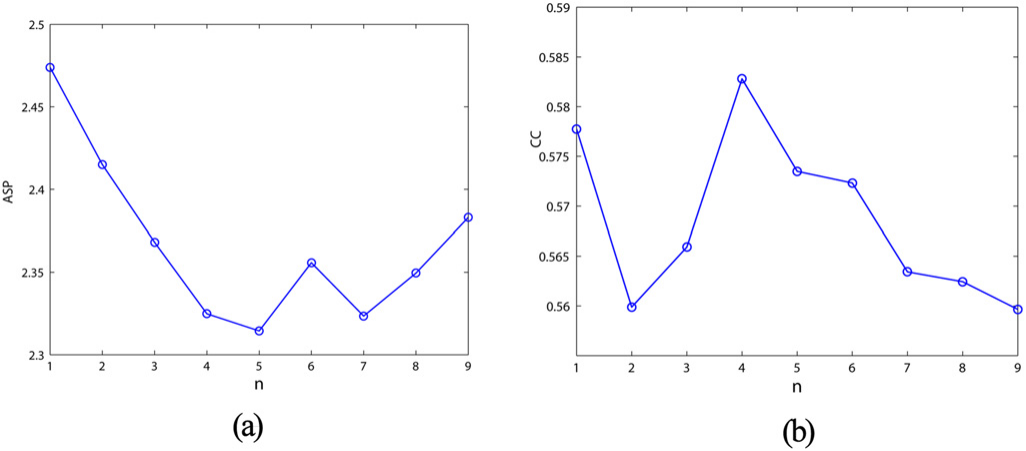
(a) ASP and (b) CC with various cluster sizes.

ASP and CC, which effectively reflect the network connectivity structure, are the basic geometric quantities based on the complex network theory [30]. Fig 10 shows the distributions of ASP and CC for the proposed DDMCESN’s network with the increasing number of pioneer nodes. Note that low ASP and large CC indicate the high level of clustering [27]. When the number of pioneer nodes is in around of 5 ASP reaches the minimum, and CC reaches the maximum. Meanwhile the DDMCESN show the optimal performance of computational capability (Fig 6). Table 4 illustrates the comparisons of the conventional ESN, MCESN, and DDMCESN. The DDMCESN and the MCESN exhibit more obvious small-network properties (i.e., a lower ASP length and a higher CC) compared to the random network. Furthermore, the DDMCESN exhibits the shortest ASP and the highest CC, which contribute to the enhancement of the system computational capability.

**Table 4:** Entropy of ESN, MCESN, and DDMCESN.

**Network**	**ASP**	**CC**
ESN	2.6902	0.037
MCESN	2.373	0.546
DDMCESN	2.359	0.562

## Conclusion

This study proposes a priori data-driven multi-cluster reservoir generation algorithm for the ESN, which is examined using the Mackey-Glass time series prediction test. The simulation results show that the proposed algorithm effectively improves the network computational capability. The prediction precision is specifically advanced during the reservoir update of the DDMCESN because of the increase of the connectivity complexity of the reservoir network. The proposed DDMCESN maintains a multi-cluster structure and possesses small-world properties. The comparative experiments show that the enhancement of the prediction precision of the DDMCESN and its standard deviation is more excellent than the traditional ESN and MCESN. The DDMCESN also has the most complex reservoir structure. Further experiments reveal that the particular configuration of the parameters of the network generation algorithm yields an optimal performance of the reservoir computing. The DDMCESN shows the best prediction performance and the largest information entropy when the number of clusters is 5 and the time window size is 0.3. The main contribution or advantage of the proposed reservoir generation algorithm is to generate an efficient multi-clustered reservoir network which can obviously promote the computing abilities of corresponding Echo state networks by an off-line updating algorithm instead of complex parameter designing.
